# Psychotic symptoms in frontotemporal dementia: a diagnostic dilemma?

**DOI:** 10.1017/S1041610214002580

**Published:** 2014-12-09

**Authors:** Maria Landqvist Waldö, Lars Gustafson, Ulla Passant, Elisabet Englund

**Affiliations:** 1 Section of Geriatric Psychiatry, Department of Clinical Sciences, Lund University, Klinikgatan 22, Lund SE-221 85, Sweden; 2 Section of Oncology and Pathology, Department of Clinical Sciences, Lund University, Lund, SE-221 85, Sweden

**Keywords:** frontotemporal dementia, psychotic symptoms, psychosis, hallucinations, delusions, paranoid ideas, neuropathology, first clinical diagnosis

## Abstract

**Background:**

Frontotemporal dementia (FTD) constitutes a spectrum of neurodegenerative disorders associated with degeneration of, predominantly, the frontal and temporal lobes. The clinical heterogeneity is evident, and early diagnosis is a challenge. The primary objectives were to characterize psychotic symptoms, initial clinical diagnoses and family history in neuropathologically verified FTD-patients and to analyze possible correlations with different neuropathological findings.

**Methods:**

The medical records of 97 consecutive patients with a neuropathological diagnosis of frontotemporal lobar degeneration (FTLD) were reevaluated. Psychotic symptoms (hallucinations, delusions, paranoid ideas), initial diagnosis and family history for psychiatric disorders were analyzed.

**Results:**

Psychotic symptoms were present in 31 patients (32%). There were no significant differences in age at onset, disease duration or gender between patients with and without psychotic symptoms. Paranoid ideas were seen in 20.6%, and hallucinations and delusions in 17.5% in equal measure. Apart from a strong correlation between psychotic symptoms and predominantly right-sided brain degeneration, the majority of patients (77.4%) were tau-negative. Only 14.4% of the patients were initially diagnosed as FTD, while other types of dementia were seen in 34%, other psychiatric disorders in 42%, and 9.2% with other cognitive/neurological disorders. The patients who were initially diagnosed with a psychiatric disorder were significantly younger than the patients with other initial clinical diagnoses. A positive heredity for dementia or other psychiatric disorder was seen in 42% and 26% of the patients respectively.

**Conclusions:**

Psychotic symptoms, not covered by current diagnostic criteria, are common and may lead to clinical misdiagnosis in FTD.

## Introduction

FTD is a clinical spectrum of neurodegenerative disorders affecting primarily the frontal and/or temporal lobes. Prominent symptoms include personality and behavioral changes as well as language disturbances. FTLD is the neuropathological umbrella term for these genetically and neuropathologically heterogeneous disorders, which as a group constitute a common cause of dementia, particularly in younger individuals. The clinical syndromes are: the behavioral variant FTD (bvFTD), the progressive aphasias semantic dementia (SD) and progressive non-fluent aphasia (PNFA) (Neary *et al.*, 1998). There is a strong association with the rapidly progressive neurological disease amyotrophic lateral sclerosis (ALS), partly attributed to overlapping genetic factors (Ferrari *et al.*, 2011). As corticobasal degeneration (CBD) and progressive supranuclear palsy (PSP) also overlap with FTD, both clinically and neuropathologically, they are often considered to be part of the FTD-complex.

Early diagnosis in FTD remains a challenge. The patients may present with a wide range of symptoms, including prominent neuropsychiatric symptoms that often mimic other psychiatric disorders. Initial psychiatric diagnoses, such as psychosis or depression, are common (Gregory and Hodges, 1996; Passant *et al.*, 2005; Woolley *et al.*, 2011).

Psychotic symptoms, including hallucinations and delusions, are known to be core symptoms in schizophrenia but are also prominent features in several neurodegenerative diseases including Alzheimer's disease and dementia with Lewy bodies (Leger and Banks, 2014). Although psychotic symptoms in FTD have been recognized for many years (Gustafson, 1987; Miller *et al.*, 1991), these symptoms are not part of any established clinical FTD criteria (Neary *et al.*, 1998; Rascovsky *et al.*, 2011). Based on previous studies, psychotic symptoms have been thought to be quite rare in FTD compared to other dementia diseases (Mendez *et al.*, 2008; Shinagawa *et al.*, 2014). Recently, however, some studies have found a higher proportion of patients with psychotic features as part of their FTD symptomatology (Snowden *et al.*, 2012; Mendez *et al.*, 2013). A recent review, based on 122 publications, concluded that the approximate prevalence of psychotic symptoms in FTD is 10% (Shinagawa *et al.*, 2014). A high prevalence of psychotic symptoms seems to be associated with specific molecular and genetic subgroups of FTD, but so far this has not been studied adequately (Shinagawa *et al.*, 2014).

Neuropathologically FTLD is divided into three subgroups, based on the major pathological protein found in neuronal and glial inclusions: tau, transactive response DNA binding protein 43 kDa (TDP-43) or fused in sarcoma (FUS) (Mackenzie *et al.*, 2010). In a few remaining patients, no specific protein pathology can be identified.

There are still relatively few studies on psychotic symptoms in neuropathologically verified FTD, and many of these are either case reports or do not differentiate between protein pathological subtypes (Shinagawa *et al.*, 2014). Various histopathological backgrounds for psychotic symptoms in FTD have been suggested. In some studies, a high prevalence of psychotic symptoms in tau-negative patients has been seen (Velakoulis *et al.*, 2009; Leger and Banks, 2014; Shinagawa *et al.*, 2014), while an equal prevalence in tau-negative and tau-positive patients has also been reported (Mendez *et al.*, 2013). Psychotic symptoms have been found to be a prominent clinical feature in a substantial number of patients with FUS-positive pathology (Urwin *et al.*, 2010).

A positive family history for dementia is generally considered to be higher in FTD compared to many other neurodegenerative diseases and has been found in around 40% of FTD patients (Rohrer *et al.*, 2009; Riedl *et al.*, 2014). So far, it has been found that autosomal dominant mutations in five genes are associated with FTD (MAPT, progranulin, C9ORF72, CHMP2b and VCP) (Riedl *et al.*, 2014). Among FTD with a known genetic background, psychotic symptoms have been found to be especially common in both progranulin and C9ORF72 carriers, sometimes even presenting years before dementia onset (Shinagawa *et al.*, 2014). One study reported a prevalence of psychotic symptoms in 25% of progranulin mutation carriers (Le Ber *et al.*, 2008), and in C9ORF72 carriers, various studies found a prevalence of around 50% or even more (Sha *et al.*, 2012; Snowden *et al.*, 2012).

Recently, it has been acknowledged that there is an overrepresentation of other psychiatric disorders, such as schizophrenia and bipolar disorder, in close relatives of FTD patients. A possible association between FTD and schizophrenia has also been discussed (Cooper and Ovsiew, 2013; Harciarek *et al.*, 2013).

There is no conclusive evidence regarding anatomical correlation of psychotic features in FTD. It has been suggested that brain regions involved in the emergence of psychotic symptoms in FTD are e.g. the thalamus and the cerebellum (Mahoney *et al.*, 2012). These brain areas also seem to be particularly affected in C9ORF72 carriers, possibly explaining the higher prevalence of psychotic symptoms in patients with this genetic mutation. Chan *et al.* (2009) found that the right temporal variant FTD exhibited more visual hallucinations (10%) than the left temporal variant FTD, SD (0%) (Chan *et al.*, 2009). It has also been proposed that there is an association between the right frontal lobe neurodegeneration and psychotic symptoms (Omar *et al.*, 2009).

The aim of the present study was to examine the prevalence and type of psychotic symptoms in neuropathologically verified FTD and to assess possible correlations with brain pathology and histopathological subclassification. In addition, the first clinical diagnosis and family history for dementia or other psychiatric disorders were analyzed.

## Methods

### Study population

The study cohort consisted of 97 consecutive patients with a neuropathological diagnosis within the FTLD-complex. The post-mortem examination had been carried out at the Department of Pathology, Lund, between 1969 and 2013. During life, all patients had been followed clinically at the Memory Clinic in Lund (previous Psychogeriatric Department). The initial clinical examinations and diagnoses were in many patients made by general practitioners, company doctors or other physicians before referral to our clinic. The study population comprised 51 females and 46 males, median age at onset was 58 (range 30–84) years and median duration was 8 (1–28) years. Age at onset was defined as the first time the symptoms attributable to the disease were noted, either by relatives or by the patient. The neuropathological patients were: tau-positive in 30.9% (9.3% Pick, 12.4% FTLD-tau, 8.2% CBD, 1.0% PSP) and tau-negative in 69.1% (TDP-43 type A 8.2%, B 39.9%, C 5.2%, D 1.0%, FUS 5.2% and FTLD with no identified protein pathology (FTLD-nipp) 10.3%). Detailed information about the study cohort, neuropathological procedures and results has recently been published (Landqvist Waldo *et al.*, 2014).

For all 97 patients, the medical records (including relevant clinical records from other hospitals and general practitioners) were systematically reviewed by two experienced MDs, first individually and then discussed at a consensus meeting. Relevant clinical information was extracted. When the reviews were made, the observers were not aware of the neuropathological details.

### Brain pathology

All patients had prior to this study, been neuropathologically examined according to standardized clinical methods at the Department of Pathology. The procedure that includes whole brain assessment covering all major areas has previously been described in detail (Landqvist Waldo *et al.*, 2014). The diagnoses and regional atrophy patterns were reached based on a combination of the original clinical-neuropathological report, of new assessment from existing hematoxylin-eosin stainings and of complementary immunohistochemical stainings. The overall severity of degeneration was assessed as mild, moderate or severe according to the same definitions as described previously. Regional pathology was assessed and noted as predominantly frontal, temporal or equally frontal and temporal (frontotemporal) as well as predominantly left, right or symmetrical degeneration. Pathology was noted as present or not present in the parietal cortex, the cerebellum, thalamus, hippocampus, basal ganglia, substantia nigra, anterior cingulate cortex (ACC), frontoinsula and the amygdala (Landqvist Waldo *et al.*, 2014).

### Psychotic symptoms

Occurrence of psychotic symptoms at onset or during the course of dementia was noted. Psychotic symptoms were noted as present, if an experienced physician (in most patients, a psychiatrist) had concluded that the patient had hallucinations, delusions or prominent paranoid ideas or if there were detailed descriptions of psychotic symptoms. The psychotic symptoms were further analyzed as to whether they consisted of hallucinations, delusions and/or paranoid ideas. Hallucinations were subdivided into visual, auditory, tactile/somatic or gustatory/olfactory hallucinations. Delusions were subdivided into three groups based on the content of false beliefs: somatic, erotomaniac or persecutory delusions (i.e. being followed, harassed, poisoned or spied on). In order to count as paranoid ideas there had to be descriptions of excessive and irrational suspiciousness or distrustfulness of other people, which reached clinical significance. Vague descriptions of slight suspiciousness or a paranoid attitude were not considered adequate to count as significant paranoid ideas. If a patient had paranoid ideas that gradually developed to persecutory delusions, the presence of both paranoid ideas and delusions was noted. The time when the psychotic symptoms occurred was recorded as during the first half (early) or the second half (late) of the dementia course for each individual.

No attempt was made to generate a diagnosis for psychotic illness according to DSM-IV, while only the psychotic symptoms documented in the medical records were noted. Delusions, hallucinations and paranoid ideas present only during delirium or confusional states were not considered.

### First clinical diagnosis

For each case, information of the first clinical diagnosis, and the time this was made, was registered. These diagnoses were categorized either as: FTD, other dementia, psychosis, depression, other psychiatric diagnosis or other diagnosis. Furthermore, bvFTD or progressive aphasia was coded as FTD, but isolated speech disturbance was categorized as other diagnosis. A diagnosis of schizophrenia, bipolar psychosis or unspecified psychosis was coded as psychosis. Personality change, stress and anxiety reactions or substance abuse were coded as other psychiatric disorder. If the patient subsequently received an FTD diagnosis, the time interval from age at onset of dementia to correct diagnosis was calculated.

### Family history of psychiatric illness

Information about family history was noted and evaluated. Family history was considered positive, if the patient had at least one first-degree relative or two or more second-degree relatives with any of the following diagnoses: (1) FTD, (2) other dementia disorders (this group may include misdiagnosed FTD patients), (3) psychotic disorders (including bipolar disorder with psychotic episodes) or (4) other psychiatric diagnoses (including depression and substance abuse).

The study was approved by the Regional Ethical Review Board in Lund, no 2014/286.

### Statistical analysis

The demographic data were described by numbers with percent or median with min/max values. Either the Fisher's exact test or the Mann–Whitney U test was used to assess possible differences between the groups. Exact calculations were performed. *P*-values below 0.05 were considered statistically significant. All statistical tests were two-sided. The statistical analyses were performed in SPSS Statistics 22 for Mac (IBM Corporation, Somers, NY, USA).

## Results

### Psychotic symptoms

Psychotic symptoms were present at some time during the course of dementia in 31 of 97 patients (32%). Basic demographics for the patient groups with and without psychotic symptoms are presented in Table 1. There were no significant differences in age at onset, disease duration or gender distribution between patients with and without psychotic symptoms.

**Table 1.** Demographic variables in FTD patients with and without psychotic symptoms

	total group	psychotic	no psychotic
	*n* = 97	symptoms *n* = 31	symptoms *n* = 66
Age at onset* (y)	58 (30–84)	56 (30–84)	59 (30–84)
Gender F/M	51/46	18/13	33/33
Disease duration* (y)	8 (1–28)	9 (1–26)	7 (2–28)

*Median (min–max).

The 31 individuals with psychotic symptoms are presented in detail in Table 2. The most common type of psychotic symptom was paranoid ideas, which was seen in 20 patients (20.6% of the total material *n* = 97). Hallucinations and delusions were equally common, present in 17 patients (17.5%). In 20 patients, the psychotic symptoms occurred early during the course of the dementia and in 11 patients only during the second half. Seven patients suffered from psychotic symptoms both early and late. In 18 of the patients more than one type of psychotic symptom was seen.

**Table 2.** FTD patients with psychotic symptoms (*n* = 31)

	gen-	age	dura-	halluci-	delu-	paranoid	predom	asym-	neuropath
patients	der	onset	tion	nations	sions	ideas	pathol	metry	diagnosis
									**Tau-pos**
1	F	60	2	e			FT		Pick
2	F	62	9	el	el	el	FT	R>L	Pick
3	F	58	4	el			T	R>L	FTLD-tau
4	F	65	7		e	e	F	R>L	FTLD-tau
5	F	68	9			l	F		FTLD-tau
6	M	56	17		e	l	F		FTLD-tau
7	M	65	9	l			F	R>L	CBD
									**Tau-neg**
8	M	55	21	l		l	F	R>L	TDP A
9	F	45	6			el	T	R>L	TDP A
10	F	50	5	l	l		F		TDP B
11	F	84	10	l			F		TDP B
12	F	58	5	el	el	el	F		TDP B
13	F	62	16		l	l	F	L>R	TDP B
14	M	68	14	e	e	e	T		TDP B
15	F	67	4		l	l	F		TDP B
16	F	52	18		e	e	FT	R>L	TDP B
17	M	46	17			el	F		TDP B
18	M	43	17	l			F		TDP B
19	F	60	2	e	e	e	F		TDP B
20	M	51	16		e	e	FT		TDP B
21	M	55	11		l	l	T	R>L	TDP C
22	M	72	4	e			F	R>L	TDP D
23	F	33	11	e	e		FT		FUS
24	F	35	7	e			F		FUS
25	M	30	8	e	e	e	FT		FUS
26	M	54	10			l	T		nipp
27	F	49	26		e	el	F	L>R	nipp
28	M	75	7	e			F		nipp
29	M	51	10		l	l	T	R>L	nipp
30	M	61	13		e	e	T	L>R	nipp
31	F	69	1	e			FT		nipp

Predom pathol = predominant pathology, Neuropath diagnosis = neuropathological diagnosis, nipp = FTLD with no identified protein pathology (FTLD-nipp), e = early, l = late, el = early and late during the disease, F = frontal, T = temporal, FT = frontotemporal.

Visual hallucinations were the most common type, seen in 14 patients (14.4%), followed by auditory hallucinations in three patients, tactile hallucinations in two patients and olfactory/gustatory hallucinations in one patient.

Out of the 17 patients with delusions, persecutory delusions were present in 11 patients, erotomania in one, somatic delusions in three and uncategorized delusions in two patients.

There was a strong correlation between psychotic symptoms and predominantly right-sided brain degeneration (*p* < 0.001). Psychotic symptoms were present in 10 of the 13 patients (76.9%) with right-sided atrophy and in 18 of the 61 (29.5%) of the symmetrical patients. Out of the 23 patients with predominantly left-sided atrophy there were only three patients (13.0%) with psychotic symptoms. However, these patients did not have any hallucinations, only delusions and paranoid ideas.

No obvious correlations were observed between psychotic symptoms and frontal or temporal pathology, nor were there any correlations between psychotic symptoms and regional degeneration in the cerebellum, thalamus, hippocampus, basal ganglia, anterior gyrus cinguli, the frontoinsular region or the amygdala.

In the total material, psychotic symptoms occurred in 7 (23.3%) of the tau-positive and 24 (35.8%) of the tau-negative cases. Among the cases with psychotic symptoms, 22.6% were tau-positive and 77.4% had tau-negative pathology. In the tau-positive patients, hallucinations were found in four, delusions in three and paranoid ideas in four cases. In the tau-negative group, there were hallucinations in 13, delusions in 14 and paranoid ideas in 16 patients.

Three out of the five (60%) patients with FUS pathology and six out of the ten (60%) patients with no identified protein pathology (FTLD-nipp) had psychotic symptoms.

### First clinical diagnosis

FTD was diagnosed during life in 78% (*n* = 76) of the patients and the median time from symptom onset to FTD diagnosis was 4 (1–15) years. Median disease duration among these 76 patients was 7.5 (2–27) years.

Clinical characteristics related to first diagnosis are presented in Table 3. Only 14 of the 97 patients (14.4%) received FTD as the first clinical diagnosis. The first clinical diagnoses for the remaining 73 patients were: other dementia in 33, psychiatric disorders in 41 (psychosis *n* = 13, depression *n* = 21, other psychiatric diagnosis *n* = 7) and other diagnoses (including MCI or speech disturbances) in 9 patients. Subsequently, all but four patients obtained a dementia diagnosis during life. The patients initially diagnosed with a psychiatric disorder were significantly younger than the patients with other first clinical diagnoses (*p* < 0.001).

**Table 3.** Clinical characteristics related to first diagnosis (*n* = 97)

first	gender	age at	time to first	ftd diagnosis	psychotic
diagnosis	m/f	onset (y)	diagnosis (y)	during life	symptoms
FTD (*n* = 14)	7/7	56.5 (42–72)	3 (1–10)	100%	35.7%
Other dementia (*n* = 33)	14/19	65.0 (45–75	2 (1–9)	66.7%	21.2%
Other (*n* = 9)	4/5	61.0 (54–75)	2 (1–6)	100%	11.1%
Psychosis (*n* = 13)	5/8	45.0 (30–62)	3 (1–10)	69.2%	69.2%
Depression (*n* = 21)	13/8	53.0 (30–84)	2 (1–6)	85.6%	23.8%
Other psychiatric (*n* = 7)	3/4	56.0 (41–84)	2 (1–4)	57.1%	57.1%

Median time to first clinical diagnosis was two years (1–10 years). Among those with an initial diagnosis of psychosis, psychotic symptoms were recorded in 69.2%. Psychotic symptoms at some stage during the course of the dementia were significantly more common among those with an initial psychiatric diagnosis (psychosis, depression, other psychiatric diagnosis) than in those without, being present in 43.9% compared to 23.2% (*p* = 0.047).

Among the patients with an initial psychiatric diagnosis 30 patients were tau-negative (45% of all tau-negative patients), and 17 of these were found to have TDP-43 type B pathology. All but one of the 13 patients with a first diagnosis of psychosis was tau-negative. All patients with FUS-pathology and seven out of ten patients with FTLD-nipp were initially given a psychiatric diagnosis.

### Family history of psychiatric illness

In three patients, there was insufficient information about family history. Among the remaining 94 patients, 41 patients (42.2%) had a positive heredity for dementia, in 27 (28.7%) there was at least one close relative with a similar dementia disease, possible FTD.

Twenty-five patients (26.6%) had a family history of other psychiatric disorders, with seven patients showing a psychotic disorder. Seven patients had at least one close relative who had committed suicide.

There was no correlation between psychotic symptoms and a positive family history of dementia. The patients with or without a family history of dementia did not differ significantly with regard to first clinical diagnosis. The FTD patients with a positive family history of psychiatric disorders were more likely to receive an initial psychiatric diagnosis than those without psychiatric disorders in the family (*p* = 0.002).

## Discussion

In this study, the prevalence and type of psychotic symptoms in a large neuropathologically diagnosed FTLD cohort was examined. Compared to many other studies a high prevalence of psychotic symptoms (32%) was seen. No major differences were found between the groups with and without psychotic symptoms with regard to the demographic variables.

This is, to our knowledge, one of the larger studies that aim to assess the prevalence of psychotic symptoms in FTD and to relate these to neuropathology. Difficulties in estimating the true prevalence of different psychotic symptoms in retrospective studies have previously been discussed, indicating a risk of both under- and overestimation (Omar *et al.*, 2009). Different inclusion criteria, such as inclusions of clinical variants (i.e. only bvFTD) or neuropathological verified patients, may explain the great variation in the prevalence of psychotic symptoms. Differences in the prevalence may also depend on the sample size, whether the samples include patients with a specific mutation (ex C9ORF72), variations in the observation time, and the instruments/scales used for the evaluation of psychotic symptoms.

In contrast to many other studies, our case selection was based on a neuropathological diagnosis within the FTLD-complex. In our patients with psychotic symptoms, 10 out of 31 were never diagnosed with FTD during life and would therefore not have been included in a clinical study on psychotic symptoms in FTD.

The clinic from which the patients were recruited was previously a psychiatric/psychogeriatric clinic and this may have influenced the awareness, and thus the reporting of psychiatric symptoms. It has previously been suggested that delusions may even be overestimated in some patients as certain behavioral manifestations typical for FTD may easily be misinterpreted as psychotic symptoms (Omar *et al.*, 2009). However, experienced psychiatrists who were accustomed to evaluating psychotic symptoms met the majority of our patients; therefore, we consider the risk of overestimation of psychotic symptoms as low.

The relatively high prevalence of psychotic symptoms in our study may, in contrast to other studies, be explained by the long follow-up time during the entire course of dementia. Psychotic symptoms may be present during a short period only or occur later during the disease. In contrast to some other studies of FTD (Gregory, 1999; Mendez *et al.*, 2008), we report symptoms from the entire course of dementia. In about 1/3 of the patients, the psychotic symptoms only occurred during the second half of dementia.

In addition to hallucinations and delusions we also included paranoid ideas, which were the most common psychotic symptoms. We attempted to separate delusions from paranoid ideas, although this distinction may be very hard to make. The concept of paranoia has been approached and discussed since the time of Kraepelin around the previous turn of the century (the 1900’s) (Kraepelin, 1919). In our study, all but four patients with paranoid ideas also had hallucinations and/or delusions. Among our patients, hallucinations were as common as delusions, in contrast to earlier reports that hallucinations are rare in FTD (Levy *et al.*, 1996).

Interestingly, we found a strong correlation between psychotic symptoms and right hemisphere predominant pathology. This has been shown earlier (Mendez *et al.*, 2008; Chan *et al.*, 2009), but not in large, neuropathologically verified materials. Among our patients, with predominantly left-sided pathology, only three patients with psychotic symptoms were found, although none of these had hallucinations. Our findings support the idea that affection of right-sided brain structures may be of importance in the emergence of psychotic symptoms in FTD. As right-sided temporal pathology has also been shown to be associated with greater socioemotional dysfunction, one may speculate that this might have implication for the appearance of psychotic symptoms (Irish *et al.*, 2013). Except for the findings regarding asymmetry, no associations between psychotic symptoms and possible affected brain areas were found. However, the fact that our assessment of regional degeneration was too blunt to reveal any associations between psychotic symptoms and specific brain pathology cannot be ruled out.

Although some studies have found differences in the prevalence between tau-positive and tau-negative patients, we did not find any significant differences between the two groups. In accordance with other studies, we found a high prevalence of psychotic symptoms in the patients with FUS pathology (Urwin *et al.*, 2010). Interestingly, in the group with no identified protein pathology (FTLD-nipp) psychotic symptoms were also highly prevalent. Future studies might reveal a common denominator in this group.

It has recently been recognized that there may be common underlying factors in FTD and psychiatric disorders such as schizophrenia and bipolar disorders. Affection of common brain networks, as well as the sharing of genetic factors, have been discussed (Zhou and Seeley, 2014). In describing schizophrenia as a dementia disorder that emerges in young adults (dementia praecox), Kraepelin was the first to recognize certain similarities (Kraepelin, 1919; Harciarek *et al.*, 2013).

Only 14.4% of the patients were initially diagnosed as FTD, but subsequently 78% received a diagnosis of FTD. The median time from symptom debut to FTD diagnosis was four years, similar to previous findings (Woolley *et al.*, 2011).

In accordance with earlier studies, we found that as many as 42% of our patients initially obtained a psychiatric diagnosis (Gregory and Hodges, 1996; Passant *et al.*, 2005; Woolley *et al.*, 2011). These patients were significantly younger than those who were initially diagnosed as FTD, other dementia or a non-psychiatric disorder. The findings that younger patients are misdiagnosed to a higher extent than elderly patients are alarming and may have devastating medical and social consequences for both the patients and their families. It highlights the great importance of educational interventions for the general practitioners, general psychiatrists and company doctors in order to recognize these patients.

Although psychiatric features are common in FTD, most patients develop only parts of psychiatric syndromes rather than the whole. Regardless of the time during the disease when the psychotic symptoms occur, the patients with psychotic symptoms are more often misdiagnosed, perhaps because these patients display symptom profiles that are not always included in current FTD criteria.

The high prevalence of psychotic symptoms in combination with the young age at onset in FUS-patients may be contributing factors explaining why all five patients in this group were initially diagnosed with either depression or psychosis.

A positive family history of dementia was seen in 42%, a prevalence similar to what has been found in other studies on heredity in FTD (Rohrer *et al.*, 2009). The patients with a positive family history of dementia were not clinically correctly diagnosed to a higher extent than those without a positive heredity. Patients with heredity for psychiatric illness were more likely to be diagnosed with a psychiatric disorder, regardless of the presence of psychotic symptoms.

The strengths and limitations of this study need to be considered. A major strength is that all patients were neuropathologically verified as FTLD. Furthermore, all patients had solid clinical records and all patients had clinical follow-ups. One limitation of this study, as of any other retrospective study, is the possibility that symptoms were present but not recognized and/or verified. This might have resulted in underreporting, thereby underestimating the prevalence of psychotic symptoms. Another limitation is that genetic mutations of interest for FTD were not screened for, as we in the majority of patients only had formalin-fixed, paraffin-embedded tissue and no blood or frozen tissue.

In conclusion, psychotic symptoms are not included in current diagnostic criteria for FTD, but need to be considered in diagnostics, treatment and care. Our results indicate a possible correlation between psychotic symptoms and right-sided brain pathology. The risk of misdiagnosis is particularly high in young patients, these individuals being diagnosed with psychiatric rather than neurodegenerative disease. The presence of psychotic symptoms in FTD is still a diagnostic dilemma and prospective clinico-pathological studies that focus on psychotic symptoms are required in order to clarify the prevalence, the etiology and to gain a clearer understanding of these phenomena.

## Conflict of interest declaration

None.

## Description of authors’ roles

M. Landqvist Waldö formulated the research questions, designed the study, collected and analyzed all data and wrote the article. L. Gustafson assisted in formulating the research questions, provided support, advice and revised the paper. U. Passant helped to design the study, supervised the clinical data collection and assisted in writing the paper. E. Englund was responsible for the neuropathological material, supervised the project and revised the paper.
